# Oligometastatic Large Cell Neuroendocrine Carcinoma of the Brain Without Radiologically Detected Primary

**DOI:** 10.14740/wjon805w

**Published:** 2014-06-25

**Authors:** Moyosore Suleiman, Michael Mullane

**Affiliations:** a Department of Hematology and Oncology, John H Stroger Jr Hospital of Cook County, Chicago, IL 60612, USA

**Keywords:** Large cell neuroendocrine carcinoma, Brain, Diagnosis

## Abstract

A 60-year-old Polish male was admitted into our hospital with complaint of right-sided lower extremity weakness. CT of head showed a left frontal 2.6 × 1.5 cm mass. Staging work-up did not show any other associated lesions in the chest or abdomen. Brain tumor was resected with histology consistent with large cell neuroendocrine carcinoma with most likely lung primary because of TTF-1 positivity. Following recovery from surgery, he had external beam radiation therapy to the brain and systemic chemotherapy with four cycles of cisplatin/etoposide. Patient is alive and doing well 6 months post diagnosis with no evidence of recurrence.

## Introduction

Large cell neuroendocrine carcinomas (LNECs) are rare malignant high-grade neuroendocrine tumors with clinical outcomes that mirror that of small cell carcinomas. LNEC of the lung is still classified under non-small lung cancers according to WHO classification but they are managed in a similar manner as small cell lung cancer (SCLC) because of poor clinical outcomes compared to other non-small cell lung cancer (NSCLC).

There are few case reports of metastatic neuroendocrine tumor of the brain without radiologically detected primary. These together with metastatic neuroendocrine tumors of other sites with unknown primary are said to arise from occult clinically undetected primary sites like lung and GI.

This case highlights the different ways of presentation of these tumors and also management challenge in terms of further systemic chemotherapy for resected oligometastatic disease with no primary lesion detected.

## Case Report

A 60-year-old Polish male presented to our hospital because of worsening right lower extremity weakness for about a month. He has had chronic low back pain ongoing for a couple of years. No problems with bowel or bladder movement were found. He has 20-pack-pear history of cigarette smoking.

Physical examination was significant for mildly reduced power in his right lower extremity with positive Babinski bilaterally.

**Figure 1:**
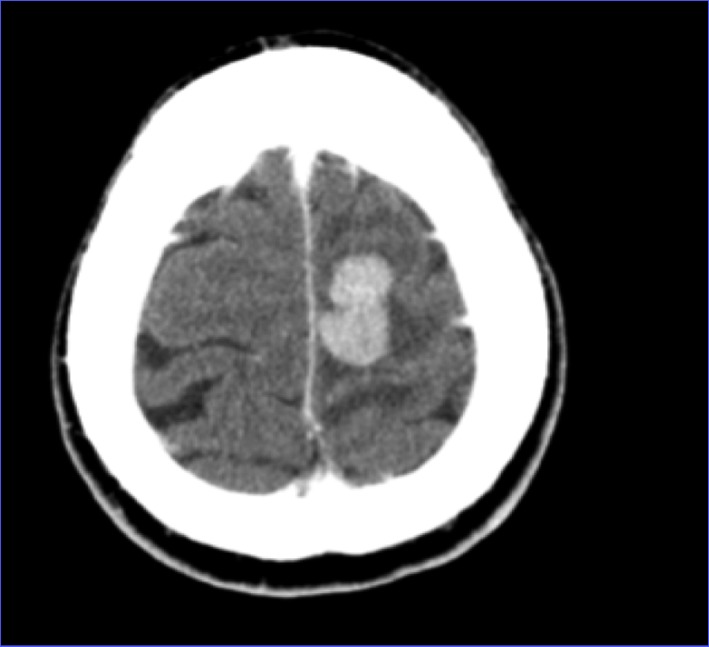
CT of head axial view showing left frontal mass.

**Figure 2:**
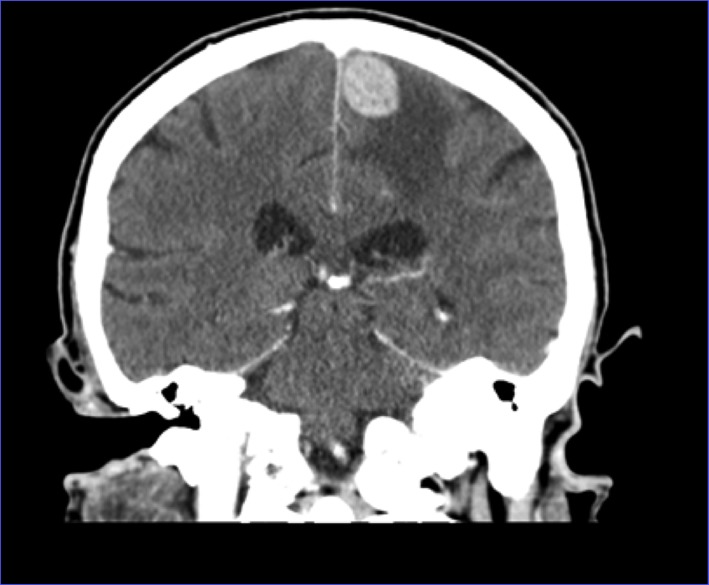
CT of head coronal view showing left frontal mass with surrounding edema.

CT of head showed a left frontal 2.6 × 1.5 cm mass (Fig. 1, 2). MRI of brain done showed a 2.7 × 1.9 × 1.7 cm lobulated oval shaped mass located in left parafalcine posterior frontal area with perilesional edema with mass effect on precentral gyrus and effacement of central sulcus.

Further staging work-up which includes CT of chest and abdomen, MRI of cervical and thoracic spine and CT of lumbar spine was unremarkable aside degenerative disease of lumbar and cervical spine. He was also noted to have an elevated PSA of 55 ng/mL as part of outpatient work-up for chronic lower back pain.

He was started on intravenous steroids and eventually had MRI-guided stereotactic left parietal craniotomy with tumor resection.

**Figure 3:**
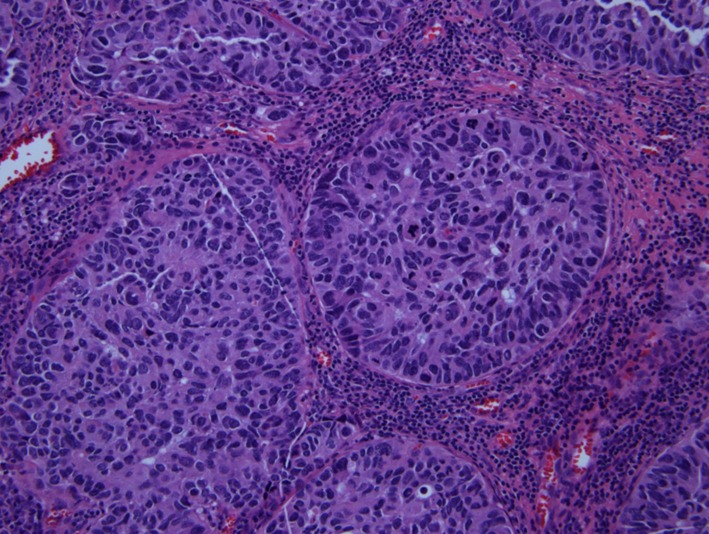
H&E showing neuroendocrine features of organoid nesting, rosette-like structures and palisading pattern.

**Figure 4:**
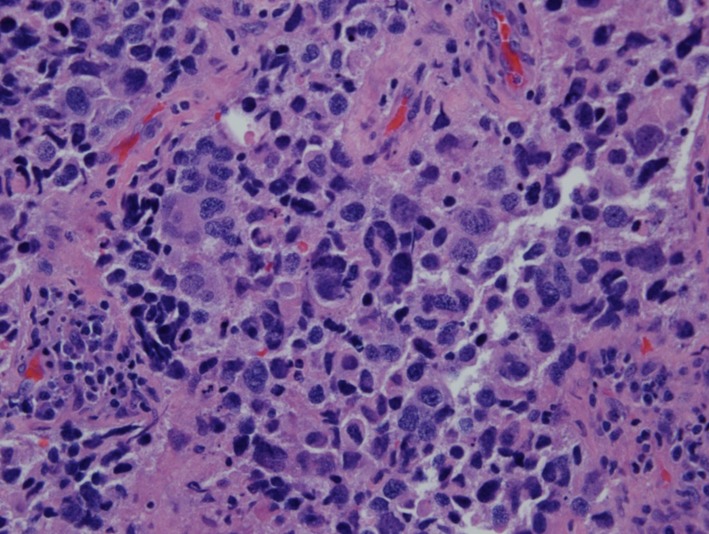
H&E (× 40) showing numerous mitotic figures.

**Figure 5:**
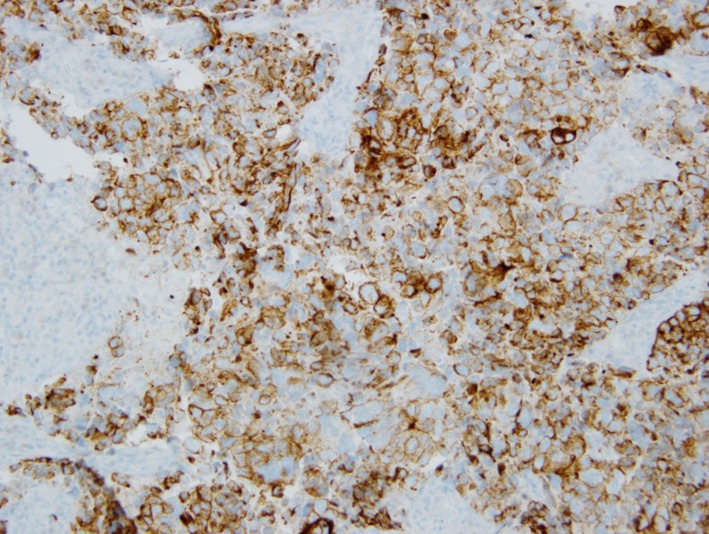
Immunohistochemical stain showing CAM 5.2 positivity.

**Figure 6:**
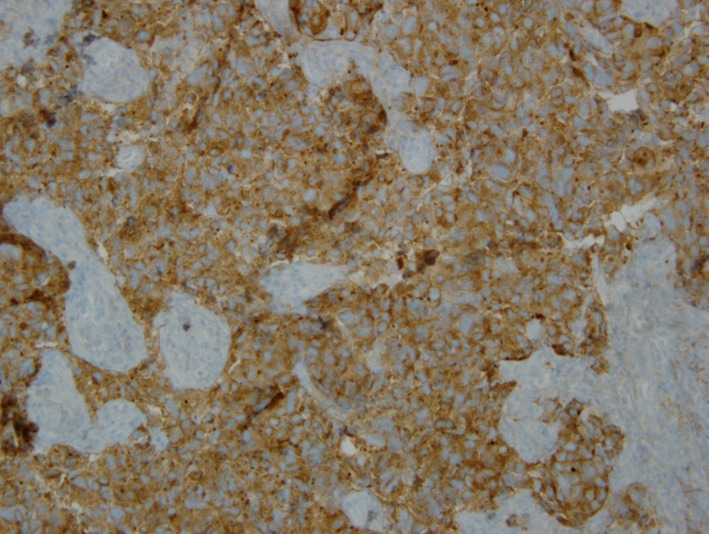
Immunohistochemical stain showing synaptophysin positivity.

**Figure 7:**
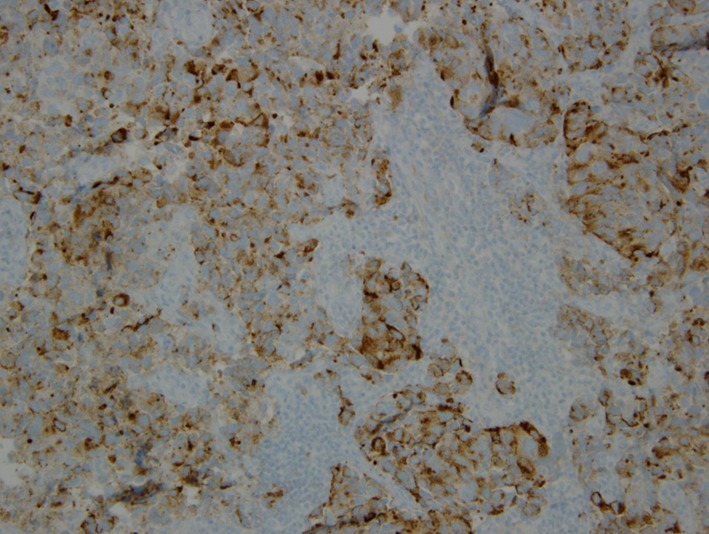
Immunohistochemical stain showing chromogranin positivity.

**Figure 8:**
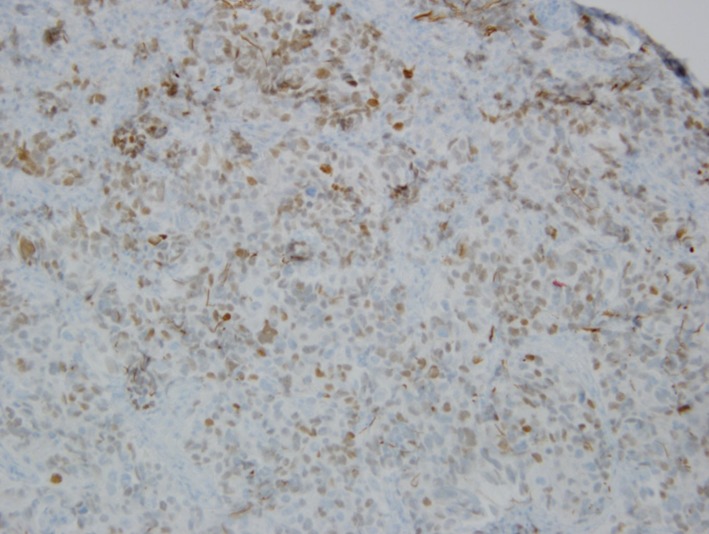
Immunohistochemical stain showing focal TTF-1 positivity.

Pathologic examination of resected tumor showed large cells growing in sheets and nests (Fig. 3) There was numerous mitosis noted with abundant necrosis indicating a high-grade tumor (Fig. 4). Immunohistochemical stains performed show that malignant cells are strongly positive for cytokeratins CAM 5.2 (Fig. 5) and CK7 and negative for cytokeratin CK20. In addition, the malignant cells are strongly positive for synaptophysin and chromogranin (Fig. 6, 7), focally positive for TTF-1 (Fig. 8) and negative for PSA, HMB45 and GFAP. This was consistent with large cell neuroendocrine metastatic carcinoma with likely lung primary.

This patient, however, did not have any lung lesion noted on imaging. He did not have any significant pulmonary symptoms either.

He had external beam radiation therapy once recovered from surgery. He also had four cycles of cisplatin/etoposide after completion of radiation therapy.

MRI of brain done 3 and 6 months post diagnosis has not shown any evidence of recurrence.

The patient also had prostate biopsy which showed prostatic adenocarcinoma Gleason 4 + 3 = 7. He was started on androgen deprivation therapy while being treated for metastatic neuroendocrine tumor and will start radiation therapy soon for prostate cancer.

## Discussion

LNEC is a rare high-grade neuroendocrine tumor that has been described in the lungs and other extra pulmonary sites [1-3]. In 1991, Travis and colleagues proposed LNEC of the lung as a distinct group of high-grade NSCLC characterized by light microscopic neuroendocrine appearance of large cells with low nuclear to cytoplasmic ratio, coarse nuclear chromatin with frequent nucleoli, high mitotic rate with frequent necrosis and neuroendocrine features by immunohistochemistry or electron microscopy [4]. LNEC of the lung is still classified under large cell carcinoma according to WHO classification [5]. Some authors have characterized the prognosis of LNEC of the lung to be intermediate between atypical carcinoid and SCLC [5]. LNEC of the lung, however, carries a worse prognosis compared to other non-small lung cancer and large cell carcinomas and is similar to small cell carcinomas [6, 7]. Clinical, histopathologic and biologic features of LNEC of the lung are more similar to other large cell carcinomas than small cell carcinomas [8]. Median survival of high-grade or poorly differentiated LNEC is about 10 months [9].

Neuroendocrine tumors of unknown primary constitute less than 5% of all cancer of unknown primary sites and 10-13% of all neuroendocrine tumors [9-11]. Most of these tumors are well-differentiated tumors [11]. Clinical behavior is variable and is dependent on tumor grade or differentiation. High-grade or poorly differentiated neuroendocrine tumors are advanced at presentation with multiple sites of metastases and rarely produce symptoms related to bioactive substances.

Few cases of metastatic neuroendocrine tumor of the brain without radiologically detected primary have been described in the literature [12-14]. Some of these cases were associated with small cell carcinomas [12, 13]. Outcome varied among patients described with rapid progression noted in a patient following resection and chemotherapy. Another patient seems to have done well 7 months post resection and chemotherapy.

Metastatic neuroendocrine tumors of unknown primary are said to be from occult clinical undetectable primary sites like lung, GI and other sites. Some patients with these kinds of tumors (isolated lymph node metastasis) constitute a distinct group with unknown primary carcinoma with relatively favorable prognosis [15].

Survival after diagnosis of cerebral metastasis is dependent on some parameters that include age, performance status, extent of extracranial disease as well as the primary diagnosis [16]. The latter is very important as some of the parameters mentioned above do not impact prognosis based on the primary malignancy [17].
